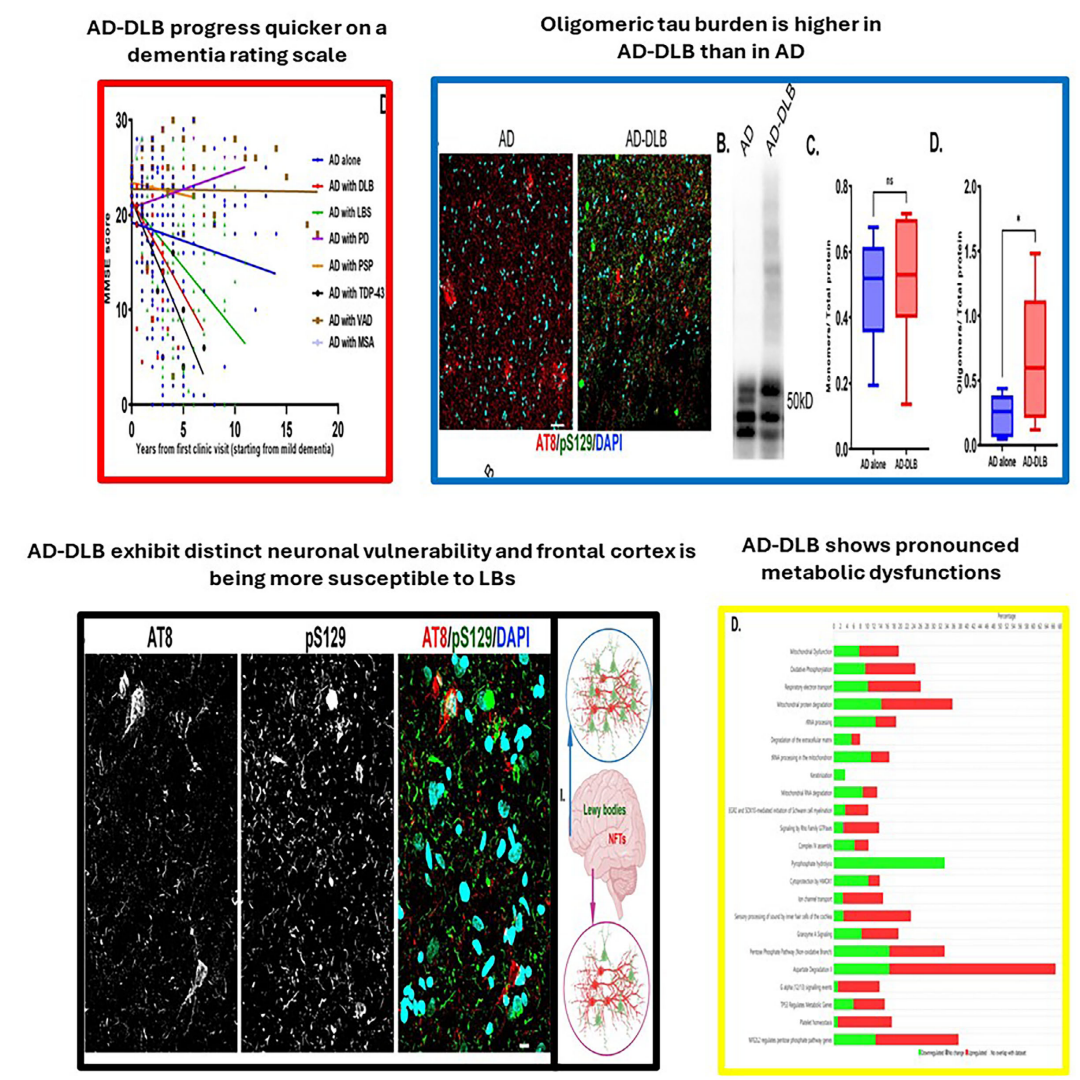# Unraveling the Heterogeneity of Dementia Progression in Alzheimer's Disease and the Impact of Lewy Body Co‐Pathology

**DOI:** 10.1002/alz70855_105655

**Published:** 2025-12-24

**Authors:** Mohammed Waseequr Rahman, Sivaprakasam Saroja

**Affiliations:** ^1^ Centre for Brain Research, Bengaluru, Karnataka, India; ^2^ Manipal Academy of Higher Education, Manipal, Karnataka, India

## Abstract

**Background:**

Alzheimer's disease (AD) is a progressive neurodegenerative disease with typical neuropathologies like Aβ and tau in the brain. Tau accumulation correlates with cognitive deterioration in AD patients. The progression of dementia in AD patients ranges from a few years to decades from the onset of the disease. Despite over a century of research in the field, it is still unclear why there is clinical heterogeneity in the dementia progression of AD patients. There can be various possibilities ranging from risk factors to overlapping neurodegenerative pathologies. Therefore, understanding the molecular complexities underlying the progression of dementia in AD patients is of prime importance in understanding the disease as a whole.

**Methods:**

We used longitudinal data of dementia progression from a U.S.A.‐based cohort of 120 patients and segregated the patients based on their co‐pathologies on survival curves. Post‐mortem temporal and prefrontal cortices of fast and slow dementia progressors were acquired from the same cohort. Biochemical, molecular, and proteomic analysis was performed using western blot, Immunohistochemistry, cellular tau seeding assays, and LC‐MS/MS.

**Results:**

Longitudinal data of dementia progression from the cohort revealed that Alzheimer's disease with dementia with Lewy bodies (AD‐DLB) progresses dementia swiftly compared to AD without any co‐pathology and AD with all other co‐pathology subgroups. AD‐DLB exhibits a high soluble *p*‐tau burden and a comparable Aβ42burden alongside additional alpha‐synuclein pathology compared to AD without any co‐pathology. Total intracellular aggregating cells are significantly high in AD‐DLB in their temporal and prefrontal cortices with distinct neuronal vulnerability for tau and alpha‐synuclein aggregation with the least co‐localization. The prefrontal cortex of AD‐DLB is more vulnerable to Lewy body aggregation than Neurofibrillary tangles (NFTs). AD‐DLB patients display unique proteomic signatures with severely affected metabolic pathways.

**Conclusion:**

Our data shed light on complex clinical and molecular intricacies within the AD continuum especially in presence of Lewy body pathology. It also highlights the urgent need for sub‐categorizing AD patients for patient‐specific and tailored clinical trials.